# Sleep Reactivity Amplifies the Impact of Pre‐Sleep Cognitive Arousal on Sleep Disturbances

**DOI:** 10.1111/jsr.70220

**Published:** 2025-10-14

**Authors:** Noof Abdullah Saad Shaif, Julian Lim, Anthony N. Reffi, Michael W. L. Chee, Stijn A. A. Massar, Ju Lynn Ong

**Affiliations:** ^1^ Centre for Sleep and Cognition, Yong Loo Lin School of Medicine National University of Singapore Singapore Singapore; ^2^ Thomas Roth Sleep Disorders and Research Center, Henry Ford Health Detroit Michigan USA; ^3^ Department of Surgery, Division of Acute Care Surgery Henry Ford Hospital Detroit Michigan USA; ^4^ Department of Psychiatry Michigan State University College of Human Medicine Grand Rapids Michigan USA

**Keywords:** multilevel models, situational insomnia, stress reactivity, stress‐related sleep disturbances, university students

## Abstract

Sleep reactivity—an individual's susceptibility to sleep disruptions due to stress—has been linked to increased insomnia risk. Investigating how sleep reactivity moderates the ‘stress → pre‐sleep arousal → sleep’ pathway may help mitigate sleep disturbances and enhance treatment outcomes. In the present study, full‐time university students without sleep disorders completed the Ford Insomnia Response to Stress Test (FIRST), which assesses sleep reactivity. From 264 students, 30 students with the lowest and 30 with the highest FIRST scores were selected for further study. They provided daily actigraphy, Pre‐sleep Arousal Scale ratings, pre‐sleep heart rate (via an ŌURA ring), and perceived stress scores over 2 weeks. Multilevel moderated mediation analyses were conducted using 800 nights of data to examine within‐ and between‐individual associations. At the within‐individual level, days with higher‐than‐usual perceived stress were associated with reduced total sleep time and increased sleep onset latency (*p*'s < 0.05). These effects were mediated by heightened pre‐sleep cognitive arousal (*p*'s < 0.05) but not moderated by the FIRST group. In contrast, between‐individual analyses revealed a significant moderation by the FIRST group (*p* < 0.05). High sleep‐reactive individuals reported significantly greater average levels of perceived stress and pre‐sleep cognitive arousal, leading to prolonged wakefulness after sleep onset (*b* = 0.123, Monte Carlo confidence interval [MCCI] = 0.006–0.292), compared to low‐reactive sleepers. Overall, on a day‐to‐day basis, both groups showed increased pre‐sleep cognitive arousal and sleep disruptions in response to elevated daily stress. However, between individuals, high sleep reactivity significantly amplified the effect of pre‐sleep cognitive (but not physiological) arousal, leading to more pronounced sleep disturbances compared to low‐reactive sleepers.

## Graphical abstract

This study investigates how sleep reactivity moderates the ‘stress‐pre‐sleep arousal‐sleep’ pathway in university students. At the within‐individual level, both high and low sleep‐reactive groups showed increased pre‐sleep cognitive arousal and sleep disruptions in response to elevated daily stress. At the between‐individual level however, high sleep reactivity significantly amplified the effect of pre‐sleep cognitive (but not physiological) arousal, leading to more pronounced sleep disturbances in high compared to low‐reactive sleepers.

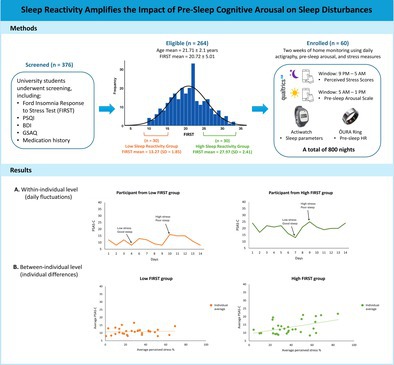

## 1 Introduction

The relationship between stress and sleep has been extensively documented (Yap et al. 2020), with chronic stress posing a significant global risk factor for the development of a myriad of mental and physical health issues downstream of sleep disturbance (American Academy of Sleep Medicine 2014). One mechanism through which stress affects sleep is pre‐sleep arousal, defined as heightened physiological and/or cognitive activity before bedtime (Dressle and Riemann 2023). Indeed, chronic stress disrupts physiological and psychological processes essential for restorative sleep, including autonomic activation and rumination (James et al. 2023). Further, persistent pre‐sleep arousal is associated with emotional dysregulation, heightened anxiety, depressive symptoms, and a greater vulnerability to chronic illnesses (Irwin et al. 2016). These data underscore the importance of identifying who is most vulnerable to pre‐sleep arousal following stressors to inform intervention strategies to interrupt the stress–arousal–sleep pathway.

A growing body of research has emphasized the role of sleep reactivity as a critical factor affecting pre‐sleep arousal (Kalmbach et al. 2018; Palagini et al. 2018). Sleep reactivity refers to an individual's vulnerability to experiencing sleep disturbances in response to stress (Drake et al. 2014). Individuals with high sleep reactivity exhibit greater pre‐sleep arousal under stress and are more likely to develop acute and chronic insomnia over time (Kalmbach et al. 2018; Kalmbach, Pillai, Arnedt, and Drake 2016; Reffi et al. 2023). These effects have been observed across various stressors, including academic stress, major life events, and laboratory‐based challenges, even in participants with good baseline sleep (Walker et al. 2022). Notably, research has pointed to pre‐sleep arousal as one mechanism by which sleep reactivity confers insomnia risk (Kalmbach et al. 2018). These constructs align with Spielman's 3P model—sleep reactivity as a predisposing vulnerability, daily stressors as precipitating events, and pre‐sleep arousal as a perpetuating mechanism (Spielman et al. 1987)—thereby linking good‐sleepers to pathways implicated in the transition from acute to chronic insomnia (Ellis et al. 2012).

Despite evidence supporting the roles of pre‐sleep arousal and sleep reactivity in stress‐related sleep disturbance, no prior studies have explored how these factors interact daily. Prior studies on stress, sleep, and sleep reactivity often rely on broad, sparse retrospective assessments (e.g., monthly or annually) (Drake et al. 2014; Jarrin et al. 2014; Kalmbach, Pillai, Arnedt, Anderson, and Drake 2016), which may overlook the more immediate and dynamic effects of daily stressors on sleep. In addition, most studies have focused on major life events, such as the loss of a loved one or job loss (Jarrin et al. 2014; Kalmbach, Pillai, Arnedt, Anderson, and Drake 2016), which, while impactful, are relatively infrequent compared to everyday stressors. For instance, daily stressors like work, school deadlines, and relational disagreements can accumulate and significantly affect sleep (Gardani et al. 2022). Furthermore, most studies in this domain are cross‐sectional or rely only on single assessments, limiting our understanding of how these processes unfold both within individuals over time and between individuals with differing vulnerabilities.

Another gap in prior research is the focus predominantly on either cognitive or physiological aspects of pre‐sleep arousal in isolation, rather than examining both simultaneously. Yet, theoretical models of insomnia, such as the hyperarousal model (Riemann et al. 2010), emphasize that both cognitive arousal (e.g., worry, rumination) and physiological arousal (e.g., increased heart rate) are central and potentially distinct pathways through which stress may interfere with sleep. These two forms of arousal, although correlated, may have differential predictive value and operate through distinct mechanisms, underscoring the need to concurrently evaluate both dimensions.

To address these gaps, the present study examined how naturally occurring fluctuations in daily stress relate to sleep, and whether pre‐sleep cognitive and physiological arousal mediate this relationship, particularly in individuals with high sleep reactivity. We investigated these processes at both the within‐individual level (day‐to‐day fluctuations) and the between‐individual level (group differences). We used a 14‐day daily design, integrating subjective stress and arousal measures with objective sleep parameters derived from actigraphy and physiological arousal indexed by wearable heart rate data. We focused on a population known to be particularly sensitive to daily stressors and sleep disruption—university students—who frequently encounter routine stressors such as academic pressures, financial concerns, and social transitions. Indeed, poor sleep among university students is prevalent and strongly linked to anxiety, depressive symptoms, and reduced academic performance (Manzar et al. 2021; Niu and Snyder 2023).

We hypothesized the following:

*At the within‐individual level, higher‐than‐usual daily stress would predict increased pre‐sleep cognitive arousal and subsequently poorer sleep outcomes—only among individuals with high sleep reactivity*.

*At the between‐individual level, individuals with high sleep reactivity would report significantly higher average levels of stress and pre‐sleep cognitive arousal, which would be associated with significantly poorer sleep outcomes, compared to low‐reactive sleepers*.

*Similar within‐ and between‐individual associations would be observed with pre‐sleep physiological arousal mediating the pathway between daily stress and sleep*.

## 2 Methods

### 2.1 Participants and Procedure

Participants were recruited via online advertisements posted on a digital platform dedicated to students studying at the National University of Singapore (NUS), if they met certain screening criteria. They were included in the study if they were full‐time NUS students aged 18–35. Exclusion criteria included: (1) any sleep disorders, as determined by the Global Sleep Assessment Questionnaire (GSAQ) (Roth et al. 2002), (2) any neurological or psychiatric conditions, including self‐reported diagnoses or suicidal ideation scores > 1 on Beck's Depression Inventory (BDI) (Beck et al. 1961), (3) use of pharmacological agents that affect wakefulness or sleep [e.g., wake‐promoting agents (Modafinil, Pitolisant), stimulants (Ritalin, Adderall), or sleep‐promoting substances (melatonin)], as assessed through the Pittsburgh Sleep Quality Index (PSQI) (Buysse et al. 1989) and medication history assessments and (4) any active illnesses, particularly infectious conditions such as flu, bronchitis, or suspected COVID‐19.

In addition, to evaluate sleep reactivity, participants completed the Ford Insomnia Response to Stress Test (FIRST) at the screening stage (Drake et al. 2004). This widely validated 9‐item scale, with scores ranging from 9 to 36, uses a four‐point Likert scale (1 = not likely, 4 = very likely) to evaluate sleep difficulties in stressful situations (Drake et al. 2004). Higher scores reflect greater sleep reactivity and increased insomnia risk. The FIRST questionnaire demonstrates high internal consistency (Cronbach's alpha = 0.83–0.88) and excellent test–retest reliability (Drake et al. 2004). Its psychometric properties have been validated across multiple studies, showing temporal stability over 6 and 12 months (Jarrin et al. 2016). These studies also proposed clinical cut‐offs, with a score of 16 or greater indicating ‘high’ sleep reactivity (Kalmbach, Pillai, Arnedt, and Drake 2016). Such cut‐offs enable researchers and clinicians to categorise individuals based on their vulnerability to stress‐induced sleep disturbances.

**FIGURE 1:**
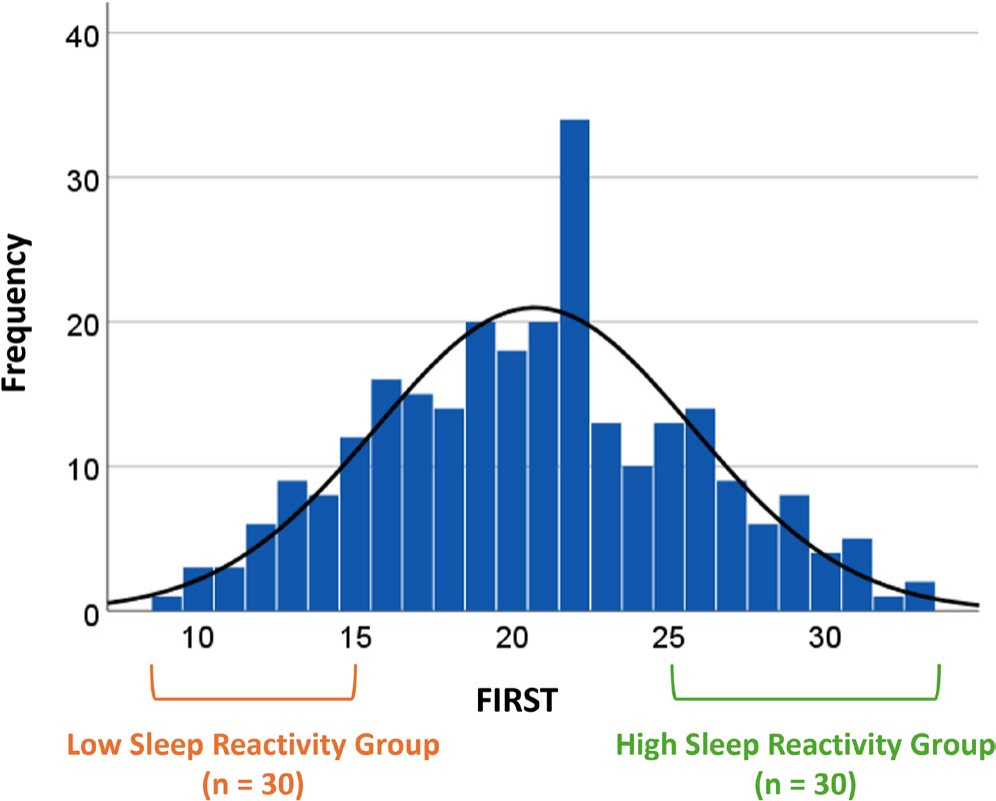
Distribution of the FIRST scores among study participants. This figure illustrates the distribution of FIRST scores within our sample (*n* = 264), delineating the classification into low and high sleep reactivity groups.

Out of 376 sign‐ups, 112 were excluded due to ineligibility, incomplete submissions, or duplicates. Of the remaining 264 eligible participants, only 60 individuals with the highest (*n* = 30) and lowest (*n* = 30) FIRST scores were selected for further study. This targeted sampling approach utilized an extreme‐groups design to classify participants into high and low sleep reactivity groups based on their FIRST questionnaire scores (Fisher et al. 2020) (Figure 1). The FIRST scores of our groups align with the established clinical cut‐off range for the FIRST questionnaire and were chosen to maximize contrast between groups while maintaining feasibility for intensive data monitoring.

These 60 participants attended an in‐person briefing session during the academic semester. At this session, participants provided consent for the study and underwent baseline measurements, including anthropometric data (height and weight) and physiological assessments (resting heart rate [HR], systolic blood pressure [SBP], and diastolic blood pressure [DBP]) using an upper arm automatic blood pressure monitor (Omron, HEM‐7121). Each physiological measurement was recorded three times at 5‐min intervals, and the average values were used for analysis (Williams et al. 2013). Participants also completed the Insomnia Severity Index (ISI), a 7‐item self‐report measure of insomnia symptom severity over the past 2 weeks [score range 0–28: no insomnia (0–7); sub‐threshold insomnia (8–14); moderate insomnia (15–21); and severe insomnia (22–28)] (Morin et al. 2011). In addition, participants were given Actiwatches for objective sleep tracking and ŌURA rings for pre‐sleep heart rate measurements, with instructions to wear them daily over 2 weeks during regular activities, except during vigorous or water‐based activities. The Institutional Review Board of the National University of Singapore (NUS‐IRB‐2022‐485) approved all research methodologies.

### 2.2 Compliance and Data Collection

Over the 2‐week study period, participants completed stress assessments before bedtime and filled out the pre‐sleep arousal scale upon waking using the Qualtrics platform (Qualtrics, USA). Responses were completed on participants' personal devices. To ensure timely completion, reminders and links to complete the stress assessments were automatically sent daily at 9 PM, with the survey remaining open until 5 AM, allowing participants to complete it before going to sleep. Similarly, reminders and links for the pre‐sleep arousal scale were sent at 5 AM, with the survey remaining open until 1 PM, allowing participants to complete it upon waking to report on pre‐sleep arousal from the night before.

**FIGURE 2:**
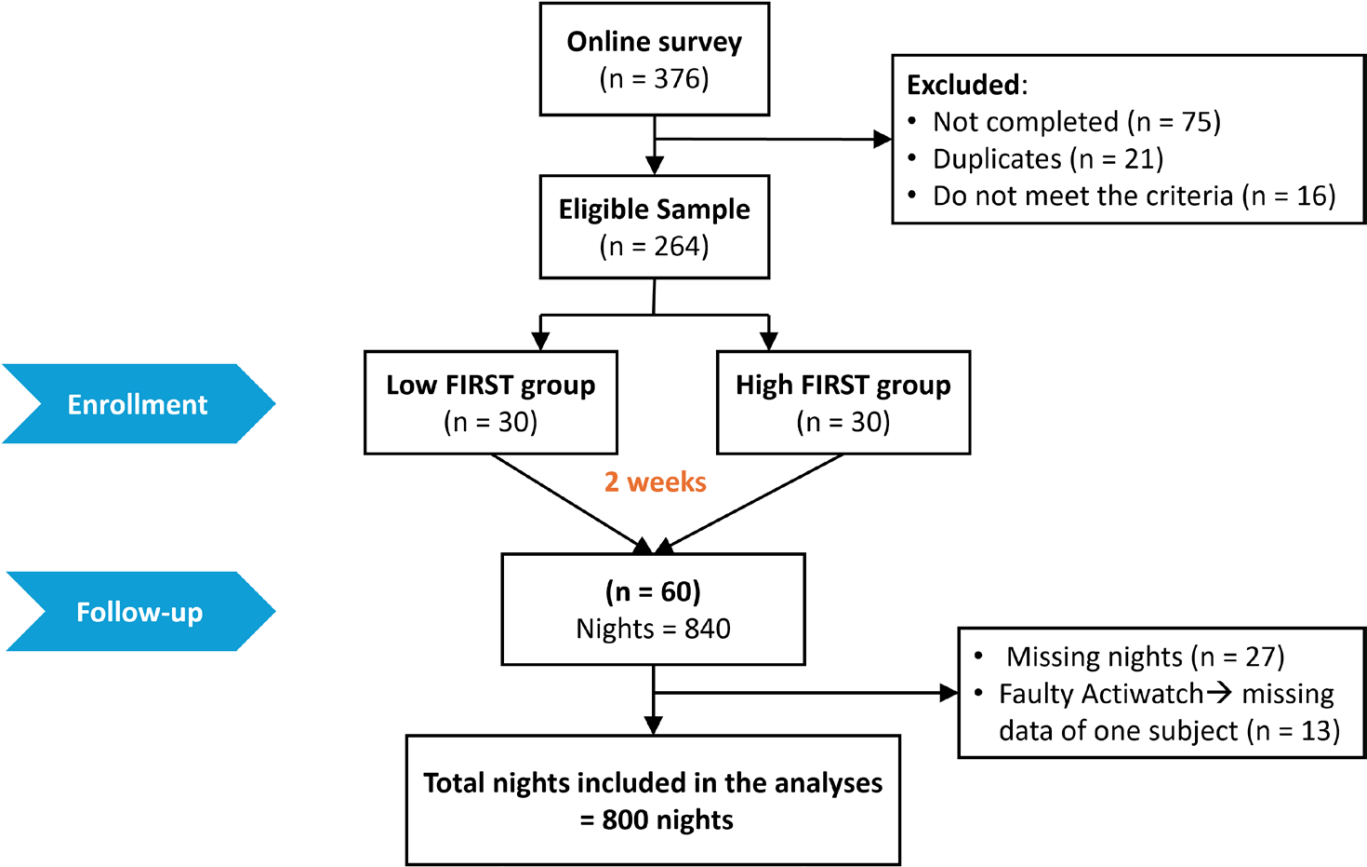
The flow chart of the study.

To ensure balanced and representative data, equal numbers of participants from the high and low FIRST groups commenced data collection each week for a 2‐week period. From the eligible pool of 60 participants, we randomly selected a batch of six participants to start each week, three in the high‐FIRST group and three in the low‐FIRST group. Students were briefed the week before their study start date, and each batch would begin and end their 14‐day protocol together. This procedure was repeated throughout the semester. All participants were studied between the start of the academic semester and the week before final examinations to avoid the potential confounding effects of heightened academic stress during finals. Additionally, distinctions were made in the analysis between sleep measurements taken on weekdays (Sunday through Thursday) and those taken on weekends (Friday and Saturday) to account for potential variations in sleep patterns. The final dataset included 800 nights of matched stress assessments, pre‐sleep arousal records, and actigraphy data, with a compliance rate of 95.23% (Figure 2).

### 2.3 Measures

#### 2.3.1 Subjective Stress Assessment

Over a 2‐week period, participants reported whether they encountered stressors in different domains each evening (Vidal Bustamante et al. 2022). They were presented with a checklist of potential stressors spanning academic (e.g., homework, grades, performance), social relationships (e.g., family, friends, significant others), and other domains (e.g., health, finances, extracurricular activities). They could select all applicable stressors or indicate if a stressor not listed was encountered. The total number of stressors for each day was computed as the sum of these sources of stress.

Participants also rated their stress intensity (perceived stress) using the item ‘On a scale from 0 to 100, indicate how stressed you are feeling right now before you sleep’, with higher scores indicating greater perceived stress. This single‐item measure of perceived stress has been utilised in previous research to assess daily stress levels (Petersen et al. 2013).

#### 2.3.2 Objective Sleep Measures

Objective sleep data were collected using actigraphy (Actiwatch Spectrum Plus, Philips Respironics Inc., Pittsburgh, PA). It uses accelerometry sensors to derive sleep–wake patterns in free‐living settings and has been validated against PSG measures in various populations (Williams et al. 2020). For 14 days, participants were required to wear the Actiwatch on their non‐dominant wrist and keep it on at all times except when engaging in sports or water‐based activity. They were instructed to press an ‘event marker’ button to indicate the times they attempted to sleep and the times they woke up. Participants were also instructed the following morning to complete the Consensus Sleep Diary online for the same period of the actigraphic recording to record their bed and wake times.

The Actiware software (version 6.0.2) was used to manually define rest periods, utilising event marker inputs, sleep diary entries, physical activity data, and light exposure readings together to accurately determine the durations of sleep and awakening. Data were de‐identified, and the rater who visually defined rest intervals was blinded to participants' FIRST group assignment. Sleep data were collected in 30‐s epochs and scored using Actiware software to calculate the total sleep time (TST), sleep onset latency (SOL), and wake after sleep onset (WASO), operationalised as follows: TST = the aggregate of minutes within a designated rest period identified as sleep by the detection algorithm, SOL = the time from the start of the designated rest interval (‘lights out’) to the first epoch classified as ‘sleep’ by the Actiware algorithm, and WASO = the total time of wakefulness occurring after sleep onset and before the final awakening in the morning.

#### 2.3.3 Subjective and Objective Pre‐Sleep Arousal Measures

##### 2.3.3.1 Self‐Reported Arousal

Subjective cognitive and somatic arousal levels were assessed daily for 14 days using the Pre‐sleep Arousal Scale (PSAS) (Nicassio et al. 1985). The PSAS is divided into two subscales: the PSAS Cognitive (PSAS‐C), focusing on nocturnal cognitive arousal (e.g., rumination or mental alertness), and the PSAS Somatic (PSAS‐S), which assesses somatic hyperarousal symptoms (e.g., irregular heartbeat or sweating). Each item is rated on a scale from 1 (not at all) to 5 (extremely), with the total score for each subscale ranging from 8 to 40, where higher scores signify greater pre‐sleep arousal levels.

##### 2.3.3.2 Objective Arousal

Objective physiological arousal levels, indexed by pre‐sleep heart rate (HR), were obtained from the ŌURA ring Gen 3 (firmware version 2.8.41; Oura, Oulu, Finland). The ring records HR in ~5‐min epochs, with multiple sub‐samples per 5‐min epoch (up to ~3). For each night, we defined ‘pre‐sleep HR’ as the mean of all valid HR samples within the 20 min immediately preceding the diary‐reported bedtime. The ŌURA ring has demonstrated strong correlations with polysomnography (PSG) for sleep metrics and with electrocardiogram (ECG) for heart rate and heart rate variability measures (Chee et al. 2021; Kinnunen et al. 2020).

### 2.4 Statistical Analyses

Multilevel modelling was used to explore the dynamics of stress, pre‐sleep arousal, sleep reactivity, and sleep over 2 weeks, allowing for an examination of both within‐individual (individuals compared to themselves) and between‐individual (individuals compared to others) associations. This approach enabled us to simultaneously investigate day‐to‐day fluctuations and individual differences in how stress, pre‐sleep arousal, and sleep outcomes are interrelated, as well as to assess whether these relationships are moderated by sleep reactivity.

Our analysis used a two‐level model: daily assessments (Level 1) nested within individuals (Level 2). Specifically, Level 1 factors captured within‐individual variations (i.e., deviations from each individual's average), examining whether individuals experiencing higher than their average stress reported greater pre‐sleep arousal and poorer sleep during the ensuing sleep period. Level 2 factors captured between‐individual variations (i.e., deviations from the group average), exploring whether participants with higher average levels of stress and pre‐sleep arousal reported poorer sleep compared to those with lower average levels of stress and pre‐sleep arousal. All analyses focused on the daily interaction between stress and sleep, with pre‐sleep arousal as a mediator and sleep reactivity as a moderator. Detailed explanations of the models and equations used to compute these associations are described elsewhere (Rockwood 2017). All coefficients were estimated as unstandardized effects to aid interpretation (i.e., minutes of sleep per 1‐unit change in stress. For example, on days when an individual feels 1 point more stressed than their own average, the models estimate *x* minutes less total sleep and/or *x* minutes more nocturnal wakefulness in that night's sleep; similarly, a 1‐point difference in individuals' 14‐day mean stress corresponds to an expected x‐minute difference in sleep outcomes.

**FIGURE 3:**
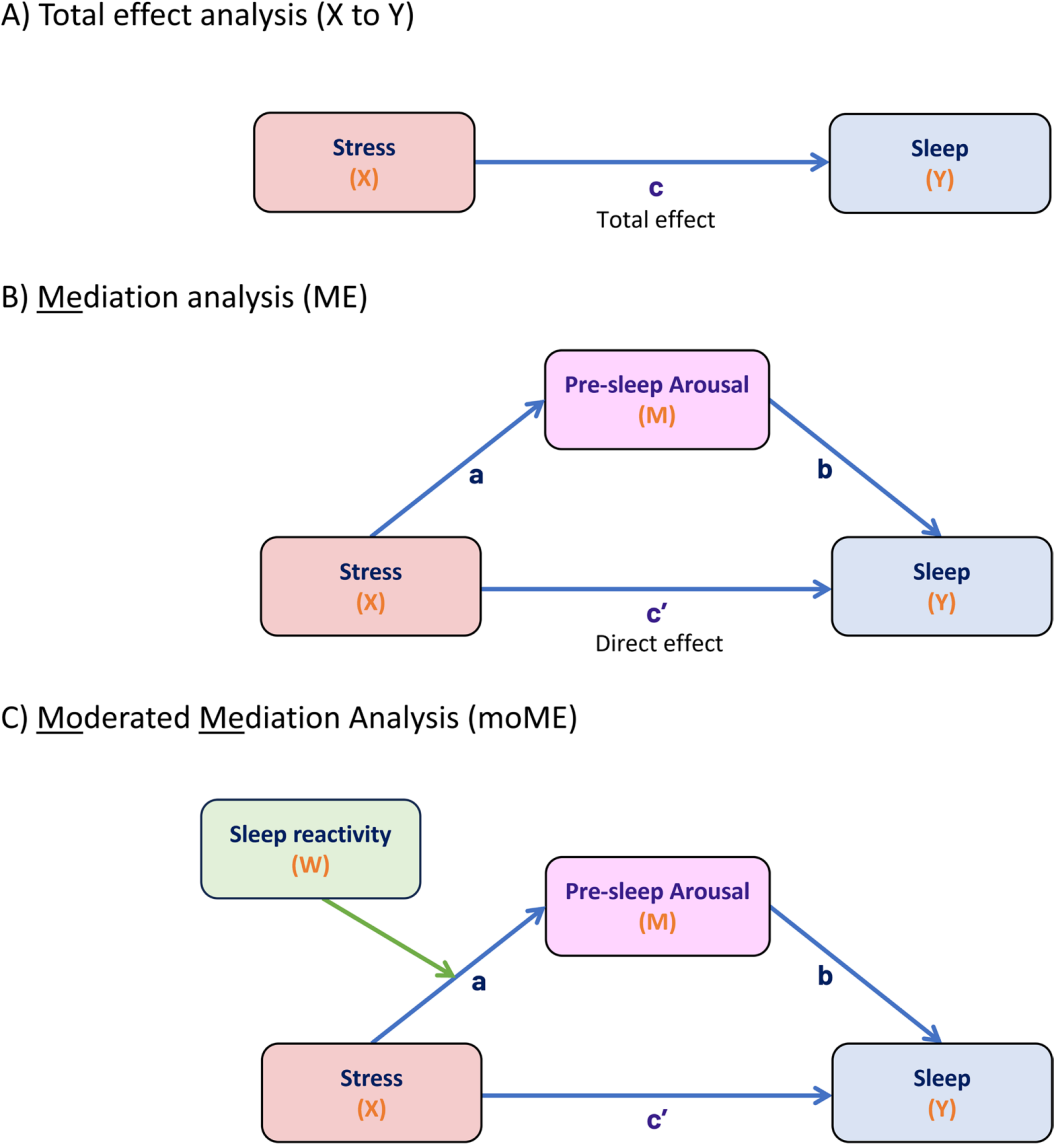
Schematic representation of analytical models used in this study representing the (a) Total effect analysis, (b) Mediation analysis, and (c) Moderated mediation analysis.

Altogether, three models were fitted to evaluate the total effect (*X* to *Y* or c‐path) of perceived stress (*X*) on three sleep parameters (*Y*): actigraphy‐measured TST, SOL, and WASO (Figure 3A). Subsequently, a mediation analysis (ME) was conducted to explore pre‐sleep arousal (M) as a mediator in the relationship between perceived stress and sleep outcomes. This model simultaneously tested the effect of perceived stress on pre‐sleep arousal (*X* to *M* or *a*‐path), pre‐sleep arousal on sleep (*M* to *Y* or *b*‐path), direct (*X* to *Y* controlling for *M* or *c*′‐path), and indirect effect of perceived stress on sleep (*X* to *M* to *Y* or *ab*‐path) (Figure 3B). To run these analyses, we used MLmed Macro Beta 2 in SPSS 26 (IBM Corp., Armonk, New York) (Hayes and Rockwood 2020; Rockwood and Hayes 2022). In this investigation, we conducted separate examinations of three distinct mediators—pre‐sleep cognitive arousal (PSAS‐C), pre‐sleep somatic arousal (PSAS‐S), and pre‐sleep heart rate (pre‐sleep HR)—to elucidate their roles in the relationship between perceived stress and sleep outcomes. Consequently, if mediation effects were present, we ran a moderated mediation analysis (moME) to examine the main hypotheses of the study, i.e., that pre‐sleep arousal acts as a mediator in the relationship between stress and sleep, with sleep reactivity group serving as a moderator (W) (Figure 3C).

This approach included calculating the moderated mediation indices using unstandardized coefficients (*b*) and Monte Carlo confidence intervals (MCCI). If the MCCI of the interaction did not contain 0, a significant moderated mediation effect could be concluded. Monte Carlo simulations were used to estimate the confidence intervals to overcome the limitations of traditional methods that might rely on assumptions about the distribution of the indirect effects, thus providing reliable and robust inference about indirect effects in the presence of moderation (Preacher and Selig 2012). All analyses included weekday/weekend distinctions as covariates to account for potential influences on the relationships under investigation. Similar to the mediation models, the moderated mediation analyses were conducted using the MLmed Macro in SPSS (Hayes and Rockwood 2020; Rockwood and Hayes 2022).

We also repeated the full analyses using Consensus Sleep Diary outcomes as subjective sleep measures (diary‐derived TST, SOL, and WASO). The 14‐day average subjective sleep parameters and the corresponding multilevel mediation and moderated‐mediation results are reported in the Supporting Information S1.

## 3 Results

### 3.1 Descriptive Statistics

#### 3.1.1 Demographic Data and Baseline Measures

Table 1 presents the means and standard deviations for demographic data and baseline measures for the low and high FIRST groups. Mean FIRST scores were significantly higher in the high FIRST group compared to the low FIRST group [high FIRST group: 27.97 (SD = 2.41) vs. low FIRST group: 13.27 (SD = 1.85); *p* < 0.001]. In addition, total PSQI scores were significantly higher in the high FIRST group (*p* < 0.001), indicating poorer self‐reported sleep quality in this group. ISI scores were also higher in the high FIRST group than in the low FIRST group (*p* = 0.003). However, there were no significant differences in the demographics and baseline physiological measures between the groups.

**TABLE 1:** Demographic data and baseline measures of low and high FIRST groups.

Baseline characteristics	Low FIRST (*n* = 30)	High FIRST (*n* = 30)	*p*
FIRST, mean (SD)	13.27 (1.85)	27.97 (2.41)	**< 0.001**
PSQI, mean (SD)	3.66 (2.08)	7.10 (2.58)	**< 0.001**
ISI, mean (SD)	5.43 (3.76)	9.30 (5.77)	**0.003**
Age (y), mean (SD)	20.83 (1.39)	21.7 (2.58)	0.112
Enrolled as			
Undergraduate	29	25	0.09
Postgraduate	1	5	
Gender			
Female	22	21	0.77
Male	8	9	
Race			
Chinese	28	23	0.07
Non‐Chinese	2	7	
Blood pressure (mmHg)			
Systolic BP	96.03 (10.34)	98.64 (13.25)	0.39
Diastolic BP	63.05 (7.67)	63.80 (6.25)	0.68
Resting heart rate (bpm)	73.72 (9.8)	77.57 (13.16)	0.20
Body mass index (kg/m^2^)	21.42 (2.97)	22.78 (4.31)	0.16

*Note:* A Pittsburgh Sleep Quality Index (PSQI) score of 5 or above indicates poor sleep quality. Insomnia Severity Index (ISI) total scores range from 0 to 28, categorised as: 0–7 no insomnia; 8–14 subthreshold insomnia; 15–21 moderate insomnia; 22–28 severe insomnia. A body mass index between 18.5 and 24.9 kg/m^2^ denotes a normal weight range. Bold indicates significance levels.

#### 3.1.2 High Reactive Sleepers Report Greater Perceived Stress Than Low Reactive Sleepers

Table 2 shows group means and standard deviations for the daily stress measures obtained. The high FIRST group reported greater average perceived stress over 14 days compared to the low FIRST group, indicating a higher level of stress perception among high FIRST participants. However, the number of stressors reported was similar between the groups (*p* > 0.05). In addition, the high FIRST group had slightly higher variability in perceived stress, as indicated by standard deviation values, but this difference did not reach statistical significance (*p* = 0.06).

**TABLE 2:** Comparison of averages and standard deviations of stress numbers, perceived stress, pre‐sleep cognitive and somatic arousal, and pre‐sleep heart rate between low and high FIRST groups.

Variables		Low FIRST	High FIRST	*p*
		Mean (SD)	Mean (SD)	
Stress measures	#Stress avg	2.53 (1.22)	3.12 (1.28)	0.72
	#Stress std	1.07 (0.37)	1.00 (0.37)	0.78
	%Stress avg	28.66 (17.71)	38.67 (19.14)	**0.04**
	%Stress std	13.02 (6.93)	16.39 (7.05)	0.06
Pre‐sleep arousal measures	PSAS‐C avg	10.88 (2.15)	13.39 (4.17)	**0.005**
	PSAS‐C std	2.53 (1.54)	3.40 (1.48)	**0.003**
	PSAS‐S avg	9.11 (1.26)	10.79 (2.78)	**0.004**
	PSAS‐S std	1.09 (0.86)	1.80 (1.01)	**0.004**
	Pre‐sleep HR avg	73.51 (5.80)	76.23 (9.39)	0.18
	Pre‐sleep HR std	7.80 (3.37)	8.20 (3.73)	0.66

*Note:* Bold indicates significance levels.

Abbreviations: avg., averages; PSAS‐C, pre‐sleep cognitive arousal; PSAS‐S, pre‐sleep somatic arousal; Pre‐sleep HR, pre‐sleep heart rate; std., standard deviations; #Stress, number of stressors; %Stress, perceived stress.

#### 3.1.3 High Reactive Sleepers Exhibit Higher Self‐Reported (But Not Objective) Pre‐Sleep Arousal

During the 14‐day study period, the high FIRST group self‐reported significantly higher mean and night‐to‐night variability in pre‐sleep cognitive and somatic arousal (PSAS) compared to the low FIRST group. Interestingly, however, objectively recorded pre‐sleep HR did not differ between the groups (*p* > 0.05) (Table 2).

#### 3.1.4 No Significant Differences in Objective Sleep Parameters Between the High and Low Reactive Sleepers

Actigraphy‐derived sleep parameters across 14 days, weekdays, and weekends revealed no significant differences between the low and high FIRST groups (*p*'s > 0.05) (Table 3).

**TABLE 3:** Comparison of averages and standard deviations of objective sleep parameters across 14 days, weekdays, and weekends for low and high FIRST groups.

Sleep parameters	Low FIRST			High FIRST		
	14 days	Weekdays	Weekends	14 days	Weekdays	Weekends
	Mean (SD)	Mean (SD)	Mean (SD)	Mean (SD)	Mean (SD)	Mean (SD)
TIB (min)	418.88 (77.58)	415.93 (78.31)	426.26 (62.58)	415.23 (73.36)	412.39 (70.14)	422.99 (81.97)
TST (min)	375.23 (73.28)	371.76 (74.14)	385.86 (61.37)	366.13 (68.10)	363.07 (66.92)	373.76 (71.64)
SE (%)	89.74 (4.32)	89.78 (4.28)	89.68 (3.91)	88.77 (4.04)	88.84 (4.05)	88.61 (3.57)
SOL (min)	14.68 (15.48)	14.11 (14.68)	15.82 (13.64)	15.78 (14.18)	15.30 (13.71)	17.04 (13.38)
WASO (min)	27.63 (10.97)	27.09 (11.25)	28.91 (8.70)	30.79 (12.98)	30.60 (12.73)	31.18 (12.57)

Abbreviations: SE, sleep efficiency; SOL, sleep onset latency; TIB, time in bed; TST, total sleep time; WASO, wake after sleep onset.

### 3.2 Multilevel Model Analyses: Pre‐Sleep Cognitive Arousal

#### 3.2.1 Multilevel Mediated Model Analysis (ME): Association Between Perceived Stress, Pre‐Sleep Cognitive Arousal, and Sleep Outcomes (*X* → *M* → *Y*; *ab*‐Path)

After identifying significant total effects in the relationship between perceived stress and sleep outcomes (*X* → *Y*, c‐path) (Table 4), we conducted multilevel mediation analyses to investigate the role of pre‐sleep cognitive arousal as a mediator in this relationship.

**TABLE 4:** Multilevel mediation analyses (ME) of perceived stress and pre‐sleep cognitive arousal on sleep outcomes: within‐ and between‐individual levels.

*X*	*M*	*Y*		*c*‐Path	*a*‐Path	*b*‐Path	*c*’‐Path	*ab*‐Path
				*b* (SE)	*b* (SE)	*b* (SE)	*b* (SE)	*b* (SE)
Within‐individual								
%Stress	PSAS‐C	Acti	TST	−0.581 (0.124)***	0.067 (0.007)***	−3.669 (0.847)***	−0.419 (0.173)*	**−0.246 (0.062)** **
			SOL	0.005 (0.026)*		1.145 (0.188)***	−0.107 (0.038)**	**0.076 (0.051)** ***
			WASO	0.091 (0.025)**		0.118 (0.151)	−0.054 (0.031)	−0.007 (0.010)
Between‐individual								
%Stress	PSAS‐C	Acti	TST	−0.943 (0.623)	0.072 (0.022)**	−1.895 (1.832)	−0.682 (0.342)	0.137 (0.145)
			SOL	0.108 (0.129)*		0.355 (0.296)	−0.011 (0.055)	0.025 (0.023)
			WASO	0.346 (0.126)**		1.287 (0.450)**	−0.179 (0.84)*	**0.093 (0.045)** *

*Note:* Bold indicates a significant effect.

Abbreviations: Acti, actigraphy; PSAS‐C, pre‐sleep cognitive arousal; SOL, sleep onset latency; %Stress, perceived stress; TST, total sleep time; WASO, wake after sleep onset.

* *p* < 0.05.

** *p* < 0.01.

*** *p* < 0.001.

At the within‐individual level (daily variability), pre‐sleep cognitive arousal was a significant mediator between stress and sleep disturbances. Days with higher‐than‐usual perceived stress were associated with reduced TST (*b* = −0.246, SE = 0.062, *p* < 0.001) and increased SOL (*b* = 0.076, SE = 0.051, *p* < 0.001), mediated by heightened pre‐sleep cognitive arousal. Specifically, a 10‐point (corresponding to the step size of the stress rating scale used [0, 10, 20, …, 100]) higher‐than‐usual stress day was associated with ~2.46 min less TST and ~0.76 min longer SOL that night via cognitive arousal, with a negligible mediated effect on WASO. This finding suggests that days with higher perceived stress predicted worse sleep the following night, and this relationship was mediated by greater pre‐sleep cognitive arousal, compared to days with lower perceived stress (Table 4).

In the between‐individual context (across individuals), we observed that pre‐sleep cognitive arousal also served as a significant mediator. Here, individuals who reported higher levels of perceived stress exhibited greater WASO (*b* = 0.093, SE = 0.045, *p* = 0.038), with this association being mediated by heightened pre‐sleep cognitive arousal (Table 4). In particular, across the sample, individuals with a 10‐point higher average stress spent 0.93 min more WASO via cognitive arousal. This indicates that individuals who typically reported higher levels of perceived stress experienced longer WASO the following night through elevated pre‐sleep cognitive arousal, compared with those who reported lower levels of perceived stress.

These findings underscore the consistent role of pre‐sleep cognitive arousal in mediating the impact of stress on sleep both within‐ and between individuals. Subsequently, we explored how individual differences in sleep reactivity could potentially moderate these relationships using a moderated mediation model (moME).

#### 3.2.2 Multilevel Moderated Mediation Model Analysis (moME): Association Between Perceived Stress, Pre‐Sleep Cognitive Arousal, Sleep Outcomes, and Sleep Reactivity (W Moderates *X* → *M*)

Our moME analysis expanded upon the multilevel mediation models by incorporating group interaction (low FIRST vs. high FIRST) as a potential moderator. Specifically, to test H1, we evaluated whether sleep reactivity moderates the relationship between stress, pre‐sleep cognitive arousal, and sleep disturbances at the within‐individual level (daily variations). We found that the mediation effect remained consistent (*b* = 0.062, SE = 0.013, *p* < 0.001) (Table 5). However, the moderated mediation indices were not significant (TST: *b* = −0.026, MCLL = −0.150, MCUL = 0.090; SOL: *b* = 0.008, MCLL = −0.027, MCUL = 0.045). This indicates that, on a daily basis, days with higher‐than‐usual perceived stress were associated with heightened pre‐sleep cognitive arousal and shorter TST and longer SOL in all individuals, irrespective of their baseline sleep reactivity levels. Thus, H1 was not supported, suggesting that sleep reactivity did not moderate this relationship on a day‐to‐day basis (Table 5).

**TABLE 5:** Multilevel moderated mediation analyses (moME) of perceived stress, pre‐sleep cognitive arousal, and sleep outcomes with group interaction: within‐ and between‐individual levels.

*W*	*X*	*M*	*Y*		*a*‐Path	*b*‐Path	*c*’‐Path	Moderated Mediation Index
					*b* (SE)	*b* (SE)	*b* (SE)	*b* (MCLL, MCUL)
Within‐individual								
FIRST Group	%Stress	PSAS‐C	Acti	TST	0.062 (0.013)***	−3.669 (0.847)***	−0.419 (0.173)*	−0.026 (−0.150, 0.090)
				SOL		1.145 (0.188)***	−0.107 (0.038)**	0.008 (−0.027, 0.045)
				WASO		—	—	—
Between‐individual								
FIRST Group	%Stress	PSAS‐C	Acti	TST	0.095 (0.044)* Group Interaction	—	—	—
				SOL		—	—	—
				WASO		1.287 (0.450)**	−0.179 (0.084)*	**0.123 (0.006, 0.292)**

*Note:* Bold indicates a significant index.

Abbreviations: Acti, actigraphy; PSAS‐C, pre‐sleep cognitive arousal; TST, total sleep time; SOL, sleep onset latency; %Stress, perceived stress; WASO, wake after sleep onset.

* *p* < 0.05.

** *p* < 0.01.

*** *p* < 0.001.

To test H2, we next investigated how individual differences in sleep reactivity influence the mediating role of pre‐sleep cognitive arousal in the relationship between stress and sleep across individuals. In contrast to the previous finding, the between‐individual analysis revealed that FIRST grouping significantly moderated the mediation effect (*b* = 0.095, SE = 0.044 x Group interaction, *p* = 0.035) (Table 5). Furthermore, the moderated mediation index was significant (*b* = 0.123, MCLL = 0.006, MCUL = 0.292), indicating that the mediated effect differed by group. Specifically, individuals with high sleep reactivity experienced significantly greater average levels of pre‐sleep cognitive arousal in response to greater perceived stress, which was associated with longer actigraphy‐measured WASO, compared to those with low sleep reactivity. Expressed per 10 points on the stress scale, the mediated WASO effect was ~1.23 min larger in the high‐FIRST group than in the low‐FIRST group. H2 was therefore supported, highlighting that individuals with high sleep reactivity are particularly vulnerable to stress‐induced pre‐sleep cognitive arousal, leading to more pronounced sleep disturbances relative to low‐reactive sleepers.

### 3.3 Multilevel Model Analyses: Pre‐Sleep Physiological Arousal

To investigate H3, similar multilevel models were conducted to evaluate whether pre‐sleep physiological arousal mediated the relationship between stress and sleep outcomes. Specifically, we examined (a) subjective somatic arousal (measured by PSAS‐S) and (b) objective heart rate (measured via the ŌURA ring) as mediators. At both within‐ and between‐individual levels, neither somatic arousal nor heart rate significantly mediated the stress–sleep relationship (*p*'s > 0.05). These findings did not align with hypothesis H3; in our sample, pre‐sleep physiological arousal—whether subjective or objective—was not a mechanism by which stress impacts objective sleep parameters.

## 4 Discussion

This study contributes to a growing body of literature on sleep reactivity by evaluating its moderating role on the ‘stress–arousal–sleep’ relationship using objective and subjective measures of pre‐sleep arousal, actigraphy‐derived sleep parameters, and stress data over 14 days, as university students went about their daily lives. In examining hypothesis H1, we found that days with higher‐than‐usual perceived stress were indeed significantly associated with poorer sleep outcomes via increased pre‐sleep cognitive arousal. However, contrary to expectations, this effect did not differ by sleep reactivity—daily fluctuations in stress elicit pre‐sleep arousal and sleep disturbances during the ensuing night, regardless of sleep reactivity. In contrast and consistent with hypothesis H2, individuals with high sleep reactivity experienced greater average levels of perceived stress and pre‐sleep cognitive arousal, which in turn contributed to more pronounced sleep disturbances—particularly longer wake after sleep onset—compared to low‐reactive sleepers.

These effects were small in size, but consistent with those observed by Yoo et al. (2023), who also reported modest daily stress–sleep reactivity effects in a 14‐day study of nurses. However, Yoo et al. found somewhat larger effects than we observed, which may be attributable to differences in sample characteristics. Specifically, their participants were nurses with night‐shift schedules, and thus likely subject to more intense stress exposure compared to our student sample. In addition, our inclusion of pre‐sleep cognitive arousal as a mediator means that we were quantifying a mechanistic pathway (stress → arousal → sleep) rather than the stress–sleep relationship, which likely contributed to the smaller indirect effects observed here.

Finally, contrary to hypothesis H3, although pre‐sleep cognitive arousal emerged as a significant mediator, neither subjective somatic arousal nor objective heart rate mediated the link between stress and sleep outcomes. This suggests that in this context, cognitive—rather than physiological—pre‐sleep arousal may be the primary mechanism through which stress influences sleep, particularly among high‐reactive individuals.

### 4.1 High‐Reactive Sleepers Reported Greater Perceived Stress and Pre‐Sleep Arousal Levels on Average but Comparable Sleep Outcomes

The high FIRST group reported significantly higher levels of perceived stress than those in the low FIRST group, despite a similar number of stressor exposures. This suggests that their heightened stress response is due to amplified stress perception rather than increased exposure, consistent with research linking perceived stress to sleep disturbances (Morin et al. 2003; Smith‐Mason and Gil‐Rivas 2019).

This observation of elevated pre‐sleep cognitive and somatic arousal in the high FIRST group aligns with studies associating these factors with high sleep reactivity (Drake et al. 2014; Fernandez‐Mendoza et al. 2010). However, no significant differences in pre‐sleep heart rate were observed, indicating that the subjective experience of somatic arousal may not always be directly reflected in measurable physiological changes, possibly due to adaptive mechanisms that buffer against stress‐induced cardiovascular changes (Brosschot et al. 2006; Reffi et al. 2022).

In addition, contrary to expectations, we found no significant differences in objective sleep parameters between low and high FIRST groups (Menghini et al. 2023). This suggests that while individuals with high sleep reactivity may perceive their sleep as disturbed (as indicated by high PSQI and ISI scores), this disturbance may not be fully reflected in averaged sleep measures. One possible explanation is that averaging sleep data over a 14‐day period could obscure transient daily variations that are more indicative of sleep reactivity. This also aligns with prior research showing that while day‐to‐day variability in actigraphy measures is a strong predictor of self‐reported sleep quality, average levels are not (Baron et al. 2017).

### 4.2 Pre‐Sleep Cognitive, but Not Physiological, Arousal Mediates the ‘Stress–Sleep’ Relationship

Our findings align with and expand upon existing literature that highlights the role of pre‐sleep cognitive arousal as a mediator in the stress–sleep relationship (Riemann et al. 2010). Our multilevel mediation analyses demonstrate that higher perceived stress was indeed associated with elevated pre‐sleep cognitive arousal, which subsequently impacts sleep outcomes—especially, reduced TST and increased SOL within‐individual, as well as longer WASO between‐individual. These results are consistent with previous work, including studies by Maskevich et al. (2020) and Kalmbach et al. (2020), which reported associations between pre‐sleep cognitive arousal and objective sleep impairments. While cognitive arousal has been closely linked to difficulties initiating sleep, PSG‐based research also indicates its association with disrupted sleep continuity (Galbiati et al. 2018; Spiegelhalder et al. 2012). Our study further contributes to this understanding by demonstrating that pre‐sleep cognitive arousal impacts sleep continuity through increased WASO at the between‐individual level. One plausible explanation is that cognitive arousal at bedtime may carry over into the night, with intrusive or ruminative thoughts resurfacing during nocturnal awakenings, thereby prolonging these wake periods (Kalmbach et al. 2020). Future studies should investigate the nature of cognitive activity during sleep maintenance difficulties to clarify this mechanism.

Interestingly, while pre‐sleep cognitive arousal significantly mediated the relationship between perceived stress and sleep, the same was not observed for pre‐sleep physiological arousal. One possible explanation is that stress levels in our sample primarily reflected minor, everyday stressors, which may not be sufficient to trigger pronounced physiological responses. It is plausible that physiological arousal emerges more prominently in response to major stressors, such as those experiencing trauma or PTSD (Reffi et al. 2023; Werner et al. 2020). Alternatively, the instruments used (i.e., self‐reported somatic pre‐sleep arousal scale and ŌURA‐derived pre‐sleep heart rate) may not fully capture the complexity of stress‐related physiological processes or may be less sensitive to detect subtle physiological changes associated with daily stress. Future studies incorporating additional physiological stress markers, such as salivary cortisol, skin conductance, or electrocardiography (ECG), are warranted to validate and extend these findings.

This divergence in the mediating roles of cognitive and physiological arousal also highlights the need for a nuanced approach to understanding and addressing the multiple dimensions of hyperarousal in sleep research. Cognitive arousal appears to have an immediate and direct impact on sleep disturbances, whereas physiological arousal may exert its effects in more complex and delayed pathways. Further exploration of the interactions between cognitive and physiological arousal is essential to gain a deeper understanding of their respective roles in sleep reactivity and sleep disturbances. In addition, our findings point to pre‐sleep cognitive arousal as a modifiable target. We therefore recommend evaluating cognitive‐behavioural therapy for insomnia (CBT‐I) and mindfulness‐based therapy for insomnia (MBTI) as interventions to reduce elevated pre‐sleep cognitive arousal in individuals with high sleep reactivity (Kalmbach et al. 2023; Reffi et al. 2023). These approaches, which focus on managing maladaptive stress responses and reframing dysfunctional thoughts and beliefs about sleep, could be especially effective for this subgroup. While evidence for improvements in objective sleep after these therapies is mixed, their established benefits on subjective sleep mean that further investigation is warranted (Mitchell et al. 2019).

### 4.3 The FIRST Questionnaire Reflects Between‐Individual Rather Than Within‐Individual Differences in Sleep Reactivity

In our within‐individual analysis (on a day‐to‐day basis), a significant association was observed across all participants between higher perceived stress and poorer sleep the following night, mediated by heightened pre‐sleep cognitive arousal. This mediation effect was unexpectedly evident in both high and low sleep reactivity groups, highlighting sleep disturbances as a natural and potentially adaptive response to stress (Ellis et al. 2012). This is consistent with the notion that short‐term sleep disruption may serve a biological adaptive function—part of the body's natural stress response—particularly in young, healthy individuals. Such temporary responses may reflect intact stress adaptation mechanisms, as long as sleep returns to baseline once the stressor is resolved (Drake et al. 2004; Petersen et al. 2013; Reffi et al. 2023).

While daily fluctuations in stress and arousal may be manageable in the short term, the cumulative burden of these responses over time may gradually erode sleep resilience and—consistent with Spielman's 3P model—contribute to the development of chronic insomnia (Drake et al. 2004; Petersen et al. 2013; Spielman et al. 1987). This maladaptive response is particularly evident in between‐individual analyses, where sleep reactivity—a predisposing factor—significantly moderated the relationship between perceived stress, pre‐sleep cognitive arousal, and sleep outcomes. Individuals with high sleep reactivity were especially susceptible to stress‐related disruptions in sleep continuity, primarily due to elevated pre‐sleep cognitive arousal. Our results also align with Perlis' neurocognitive model, which posits that pre‐sleep worry and rumination amplify cognitive arousal, thereby sustaining cortical hyperarousal and maintaining insomnia (Perlis et al. 1997). Heightened cortical activity interferes with normal sleep processes by impeding transitions into deeper NREM sleep and enhancing the brain's processing of sensory input, which in turn elevates vigilance and undermines disengagement from external stimuli (Dressle et al. 2023). This underscores how heightened sleep reactivity amplifies the adverse effects of stress and pre‐sleep cognitive arousal on sleep, as reflected in longer actigraphy‐derived WASO.

## 5 Study Strengths and Limitations

One of the key strengths of this study is the use of multilevel modelling, which enabled simultaneous examination of both within‐ and between‐individual associations, offering a nuanced understanding of how daily stress and pre‐sleep arousal interact to influence sleep outcomes over time. By focusing on minor, everyday stressors—rather than infrequent, high‐impact events—the study captures the ecologically valid experiences that more accurately reflect naturally occurring contributors to sleep disturbances. Another important strength is the concurrent examination of both cognitive and physiological components of pre‐sleep arousal, providing a more comprehensive understanding of the hyperarousal processes implicated in insomnia. This was further enhanced by the integration of subjective and objective measures of pre‐sleep arousal, alongside objective sleep metrics, strengthening the methodological rigour and validity of the findings. Finally, by leveraging longitudinal data from wearable devices, the study demonstrates the feasibility of scalable, non‐invasive tools for monitoring stress and sleep in naturalistic settings. This approach not only enhances ecological validity but also offers a practical pathway for future research on early detection and personalised intervention for sleep disturbances.

Despite these strengths, the study has some limitations. The sample comprised predominantly young, female, Chinese university students, which may limit the generalizability of the findings to more diverse populations. Furthermore, the exclusion of individuals with clinically diagnosed sleep disorders or those taking sleep‐affecting medications may restrict the broader applicability of the results to clinical or older adult populations.

Another methodological limitation is the timing of the Pre‐Sleep Arousal Scale (PSAS). In this study, the PSAS was administered the morning after sleep, whereas our objective arousal marker (pre‐sleep heart rate) was recorded directly during the pre‐sleep window. We adopted morning administration to minimise potential measurement‐related reactivity at bedtime; prompting participants at lights‐out can itself elevate cognitive/somatic arousal, introduce device/light exposure, and potentially contaminate the pre‐sleep period. We acknowledge that morning administration makes the PSAS a retrospective measure that may be influenced by the intervening sleep experience. Future studies could mitigate this by collecting PSAS ratings at bedtime via low‐light, low‐burden assessment and synchronising the timing of subjective and objective indices to better align constructs.

## 6 Conclusion and Future Directions

In conclusion, on a day‐to‐day basis, days with higher‐than‐usual perceived stress were associated with worse sleep outcomes the following night through increased pre‐sleep cognitive arousal, regardless of sleep reactivity levels. This suggests that both groups maintain intact stress adaptation mechanisms. However, between individuals, those with high sleep reactivity reported significantly higher average levels of perceived stress and pre‐sleep cognitive arousal, leading to more pronounced sleep disturbances compared to low‐reactive sleepers.

Our findings suggest that individuals with high sleep reactivity might benefit from interventions targeting their heightened pre‐sleep cognitive arousal and stress sensitivities. Delivering such interventions early to individuals with high sleep reactivity and acute sleep disturbances may provide the most significant advantages by preventing the progression to chronic insomnia.

Future research should focus on replicating these findings in more diverse populations, and on investigating the roles of resilience, social support, and other protective factors in mitigating the impact of stress and pre‐sleep cognitive arousal on individuals with high sleep reactivity. Additionally, the potential to normalize sleep reactivity through interventions such as CBT‐I and MBTI presents an exciting avenue for future research. Understanding the mechanisms through which these interventions modulate sleep reactivity could lead to more effective treatments for insomnia and sleep disturbances, ultimately improving sleep and overall well‐being. [Correction added on 07 November 2025, after first online publication: The interpretations of coefficients in Tables 4 and 5 were provided in 10‐point intervals instead of 1‐point intervals so the decimals have been shifted accordingly throughout the text.]

## Author Contributions

N.A.A.S. led the conceptualization, conducted the formal analysis, and drafted the original manuscript. J.L., A.N.R., and M.W.L.C. contributed to the review and editing of the manuscript. S.A.A.M. and J.L.O. were responsible for conceptualization, supervision, and manuscript review and editing.

## Conflicts of Interest

Dr. Michael WL Chee is on the Medical Advisory Board of Oura Health, but this work was independent of any commercial interest. The authors declare no conflicts of interest.

## Supporting information


**Table S1:** Comparison of averages and standard deviations of subjective sleep parameters across 14 days, weekdays, and weekends for low and high FIRST groups
**Table S2:** Multilevel mediation analyses (ME) of perceived stress and pre‐sleep cognitive arousal on subjective sleep outcomes: within‐ and between‐individual levels
**Table S3:** Multilevel moderated mediation analyses (moME) of perceived stress, pre‐sleep cognitive arousal, and subjective sleep outcomes with group interaction: within‐ and between‐individual levels.

## Data Availability

The data that support the findings of this study are available from the corresponding author upon reasonable request.
