# Supplementary material for: Sleep Reactivity Amplifies the Impact of Pre‐Sleep Cognitive Arousal on Sleep Disturbances
Source: J Sleep Res. 2025 Oct 14;35(3):e70220. doi: 10.1111/jsr.70220 (PMC12927662; doi:10.1111/jsr.70220)
Supplement: Supplementary file 1 — Table S1: Comparison of averages and standard deviations of subjective sleep parameters across 14 days, weekdays, and weekends for low and high FIRST groups Table S2: Multilevel mediation analyses (ME) of perceived stress and pre‐sleep cognitive arousal on subjective sleep outcomes: within‐ and between‐individual levels Table S3: Multilevel moderated mediation analyses (moME) of perceived stress, pre‐sleep cognitive arousal, and subjective sleep outcomes with group interaction: within‐ and between‐individual levels. [file JSR-35-e70220-s001.docx]

**Table S1**. **Comparison of Averages and Standard Deviations of Subjective Sleep Parameters across 14 Days, Weekdays, and Weekends for Low and High FIRST Groups.**

| **Sleep Parameters** | **Low FIRST** | | | **High FIRST** | | |
| --- | --- | --- | --- | --- | --- | --- |
|  | **14 DAYS**  Mean (SD) | **WEEKDAYS**  Mean (SD) | **WEEKENDS**  Mean (SD) | **14-DAYS**  Mean (SD) | **WEEKDAYS**  Mean (SD) | **WEEKENDS**  Mean (SD) |
| **TIB (min)** | 420.27 (79.91) | 417.52 (77.53) | 427.17 (62.85) | 414.99 (73.3) | 412.13 (70.02) | 422.99 (81.97) |
| **TST (min)** | 404.39 (76.46) | 401.49 (76.98) | 411.95 (62.40) | 394.52 (74.08) | 392.61 (71.29) | 400.28 (81.25) |
| **SE (%)** | 96.19 (3.05) | 96.11 (2.96) | 96.45 (2.34) | 95.01 (3.78) | 95.16 (3.46) | 95.66 (2.9) |
| **SOL (min)** | 10.67 (8.98) | 10.93 (8.81) | 9.84 (6.56) | 13.53 (10.58) | 13.05 (8.57) | 14.66 (11.86) |
| **WASO (min)** | 5.11 (6.98) | 4.88 (6.12) | 5.54 (6.12) | 6.93 (8.23) | 6.46 (7.27) | 8.05 (7.75) |

TIB, Time in Bed; TST, Total Sleep Time; SE, Sleep Efficiency; SOL, Sleep Onset Latency; WASO, Wake After Sleep Onset.

**Table S2.** **Multilevel Mediation Analyses (ME) of Perceived Stress and Pre-sleep Cognitive Arousal on Subjective Sleep Outcomes: Within- and Between-Individual Levels.**

| **X** | **M** | **Y** | | **c-path**  b (SE) | **a-path**  b (SE) | **b-path**  b (SE) | **c’-path**  b (SE) | **ab-path**  b (SE) |
| --- | --- | --- | --- | --- | --- | --- | --- | --- |
| **Within-Individual** | | | | | | | | |
| %Stress | PSAS-C | Diary | TST | **- 0.644 (0.130)***** | **0.066 (0.007)***** | **- 4.539 (0.891)***** | **- 0.517 (0.182)**** | **- 0.302 (0.067)***** |
|  |  |  | SOL | **0.025 (0.021)*** |  | **1.651 (0.133)***** | - 0.048 (0.027) | **0.110 (0.0147) ***** |
|  |  |  | WASO | **0.019 (0.018)*** |  | **0.451 (0.122)**** | - 0.022 (0.025) | **0.030 (0.008)**** |
| **Between-Individual** | | | | | | | | |
| %Stress | PSAS-C | Diary | TST | - 0.913 (0.619) | 0.073  (0.022)** | - 1.219 (1.896) | - 0.625 (0.353) | 0.089 (0.148) |
|  |  |  | SOL | **0.266 (0.100)**** |  | **1.066 (0.300)**** | - 0.066 (0.056) | **0.078 (0.033)*** |
|  |  |  | WASO | **0.179 (0.086)*** |  | 0.475 (0.290) | - 0.059 (0.054) | 0.035 (0.024) |

%Stress, perceived stress; PSAS-C, pre-sleep cognitive arousal, TST, Total Sleep Time; SOL, Sleep Onset Latency; WASO, Wake After Sleep Onset. * *p* < 0.05, ** *p* < 0.01, or *** *p* < 0.001 levels.

**Table S3.** **Multilevel Moderated Mediation Analyses (moME) of Perceived Stress, Pre-Sleep Cognitive Arousal, and Subjective Sleep Outcomes with Group Interaction: Within- and Between-Individual Levels.**

| **W** | **X** | **M** | **Y** | | **a-path**  b (SE) | **b-path**  b (SE) | **c’-path**  b (SE) | **Moderated Mediation Index**  b (MCLL, MCUL) |
| --- | --- | --- | --- | --- | --- | --- | --- | --- |
| **Within-Individual** | | | | | | | | |
| FIRST | %Stress | PSAS-C | Diary | TST | 0.060 (0.011)*** | **- 4.539 (0.891)***** | **- 0.517 (0.182)**** | - 0.045 (-0.184, 0.083) |
|  |  |  |  | SOL |  | **1.651 (0.133) ***** | - 0.048 (0.027) | 0.016 (-0.029, 0.063) |
|  |  |  |  | WASO |  | **0.451 (0.122)**** | - 0.022 (0.025) | 0.004 (-0.008, 0.019) |
| **Between-Individual** | | | | | | | | |
| FIRST | %Stress | PSAS-C | Diary | TST | 0.093 (0.043)*  Group Interaction | — | — | — |
|  |  |  |  | SOL |  | **1.069 (0.300)**** | - 0.066 (0.056) | **0.100 (0.007, 0.225)** |
|  |  |  |  | WASO |  | — | — | — |

%Stress, perceived stress; PSAS-C, pre-sleep cognitive arousal, TST, Total Sleep Time; SOL, Sleep Onset Latency; WASO, Wake After Sleep Onset. **p* < 0.05, ** *p* < 0.01, or *** *p* < 0.001 levels.
